# Supplementary material for: Machine Learning Approaches for the Prediction of Postoperative Major Complications in Patients Undergoing Surgery for Bowel Obstruction
Source: J Pers Med. 2024 Oct 8;14(10):1043. doi: 10.3390/jpm14101043 (PMC11508771; doi:10.3390/jpm14101043)
Supplement: Supplementary file 1 [file jpm-14-01043-s001.zip › jpm-3153309-supplementary.pptx]

## Slide 1
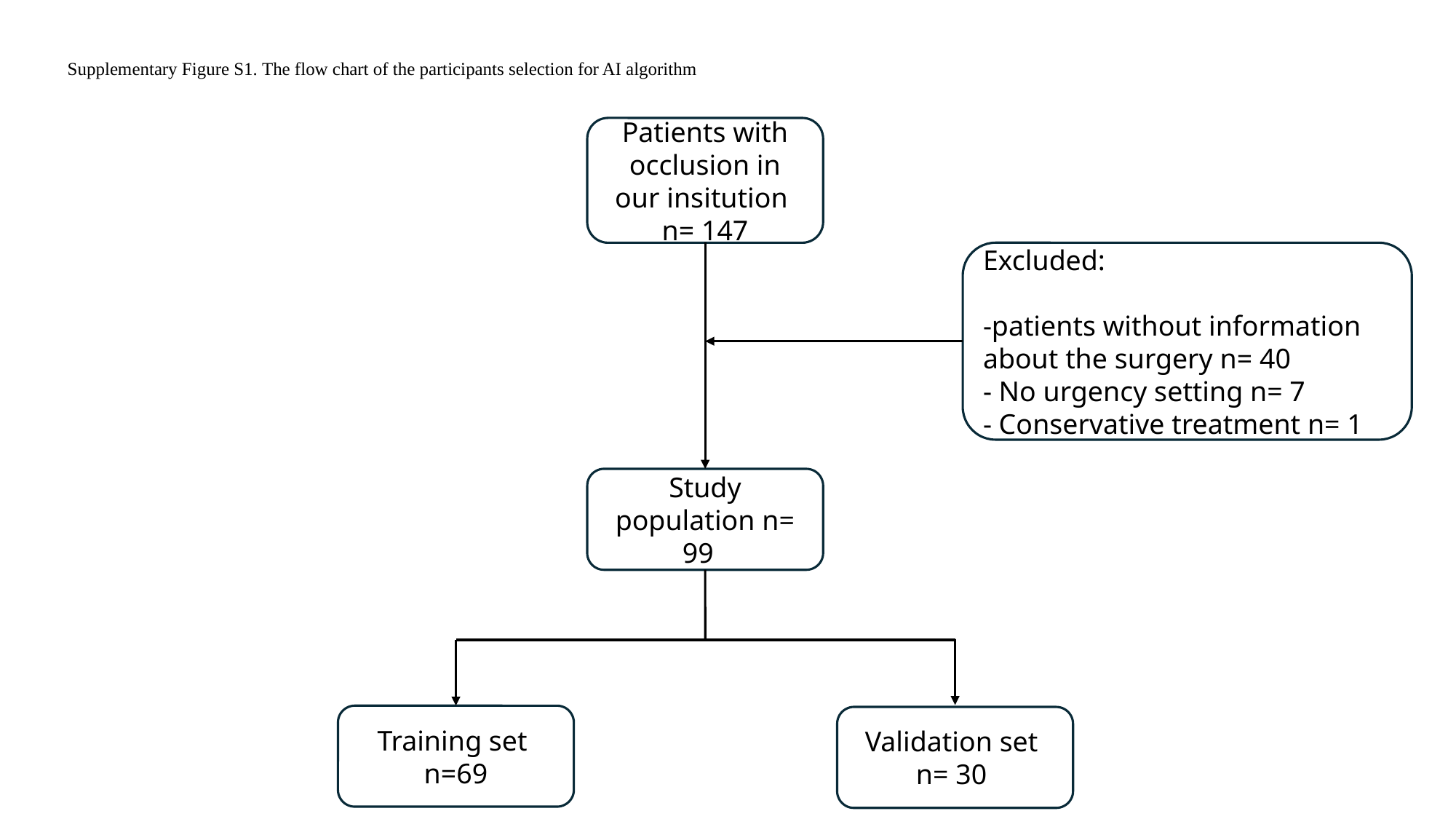

Supplementary Figure S1. The flow chart of the participants selection for AI algorithm
Patients with occlusion in our insitution
n= 147
Excluded:
-patients without information about the surgery n= 40
- No urgency setting n= 7
- Conservative treatment n= 1
Study population n= 99
Training set
n=69
Validation set
n= 30
